# Supplementary material for: UPLC-MS/MS Identification and Quantification of Withanolides from Six Parts of the Medicinal Plant Datura Metel L
Source: Molecules. 2020 Mar 11;25(6):1260. doi: 10.3390/molecules25061260 (PMC7144020; doi:10.3390/molecules25061260)
Supplement: Supplementary file 1 [file molecules-25-01260-s001.pdf]

Supporting Information

UPLC-MS/MS Identification and Quantification of Withanolides from Six Parts of the Medicinal Plant *Datura Metel* L.

Yang Shi Hui [1†], Liu Yan [1†], Wang Qi [2], Sun Yan Ping [1], Guan Wei [1], Liu Yuan [1], Yang Bing You [1*], and Kuang Hai Xue[1*]

Affiliations:

[1] *Key Laboratory of Chinese Materia, Heilongjiang University of Chinese Medicine, Ministry of Education, Harbin 150040, China*

[2] *Department of Medicinal Chemistry and Natural Medicine Chemistry, College of Pharmacy, Harbin Medical University, Harbin 150040, China*

[*] *Corresponding authors. E-mail address: ybywater@163.com (Yang Bing You), hxkuang@yahoo.com (Kuang Hai Xue).*

Table of Contents

Figure S1. The BPC graphs of six parts of *Datura metel* L. *(D. metel* L.*)*: 1A for ESC+, 1B for ESC-.

Figure S2. The distribution of 22 withanolides in six parts of *D. metel* L. : Baimantuoluoside H (9), Daturafoliside K (12), Baimantuoluoside B (13), Daturafoliside B (16), Daturafoliside A (23), 5*α*,12*α*,27-trihydroxy-(20S,22R) -6*α*,7*α*-epoxy-1-oxowitha-2,24-dienolide (26), Daturafoliside O (30), Daturametelin J (32), Daturafoliside Q (33), Daturafoliside D (36), Daturafoliside S (46), Daturafoliside I (49), 7*α*,27-dihydroxy-(20S,22R)-1-oxowitha-2,5,24-trienolide-27-O-β-D-glucopyranosy (53), Daturataturin B (55), Daturafoliside Y (59), 7*α*,27-dihydroxy-(20S, 22R)-7-methoxy-1-oxowitha-3,5,24-trienolide-27-O-β-D-glucopyranosy (63). Daturametelin I (64), Daturataturin A (65), 7*α*,27-dihydroxy-1-oxowitha-2,5,24-trienolide (69), Daturafoliside F (72), Daturafoliside X (78), Daturametelin A (80).

Figure S3. The total withanolides contents of different extraction conditions: extraction methods (A), extraction solvents (B), extraction times (C) and extraction repeats (D).

Figure S4. The MRM chromatograms of 22 bioactive withanolides: Baimantuoluoside H (9), Daturafoliside K (12), Baimantuoluoside B (13), Daturafoliside B (16), Daturafoliside A (23), 5*α*,12*α*,27-trihydroxy-(20S,22R) -6*α*,7*α*-epoxy-1-oxowitha-2,24-dienolide (26), Daturafoliside O (30), Daturametelin J (32), Daturafoliside Q (33), Daturafoliside D (36), Daturafoliside S (46), Daturafoliside I (49), 7*α*,27-dihydroxy-(20S,22R)-1-oxowitha-2,5,24-trienolide-27-O-β-D-glucopyranosy (53), Daturataturin B (55), Daturafoliside Y (59), 7*α*,27-dihydroxy-(20S, 22R)-7-methoxy-1-oxowitha-3,5,24-trienolide-27-O-β-D-glucopyranosy (63). Daturametelin I (64), Daturataturin A (65), 7*α*,27-dihydroxy-1-oxowitha-2,5,24-trienolide (69), Daturafoliside F (72), Daturafoliside X (78), Daturametelin A (80).

Table S1. The contents of 22 bioactive withanolides in *Datura metel* L. *(D. metel* L.*)*.

Table S2. The total withanolides contents of different extraction conditions for *D. metel* L..

Table S3. Information of *D. metel* L. materials.

Table S4. UPLC-Q-TRAP-MS/MS detection parameters for 22 bioactive withanolides in *D. metel* L..

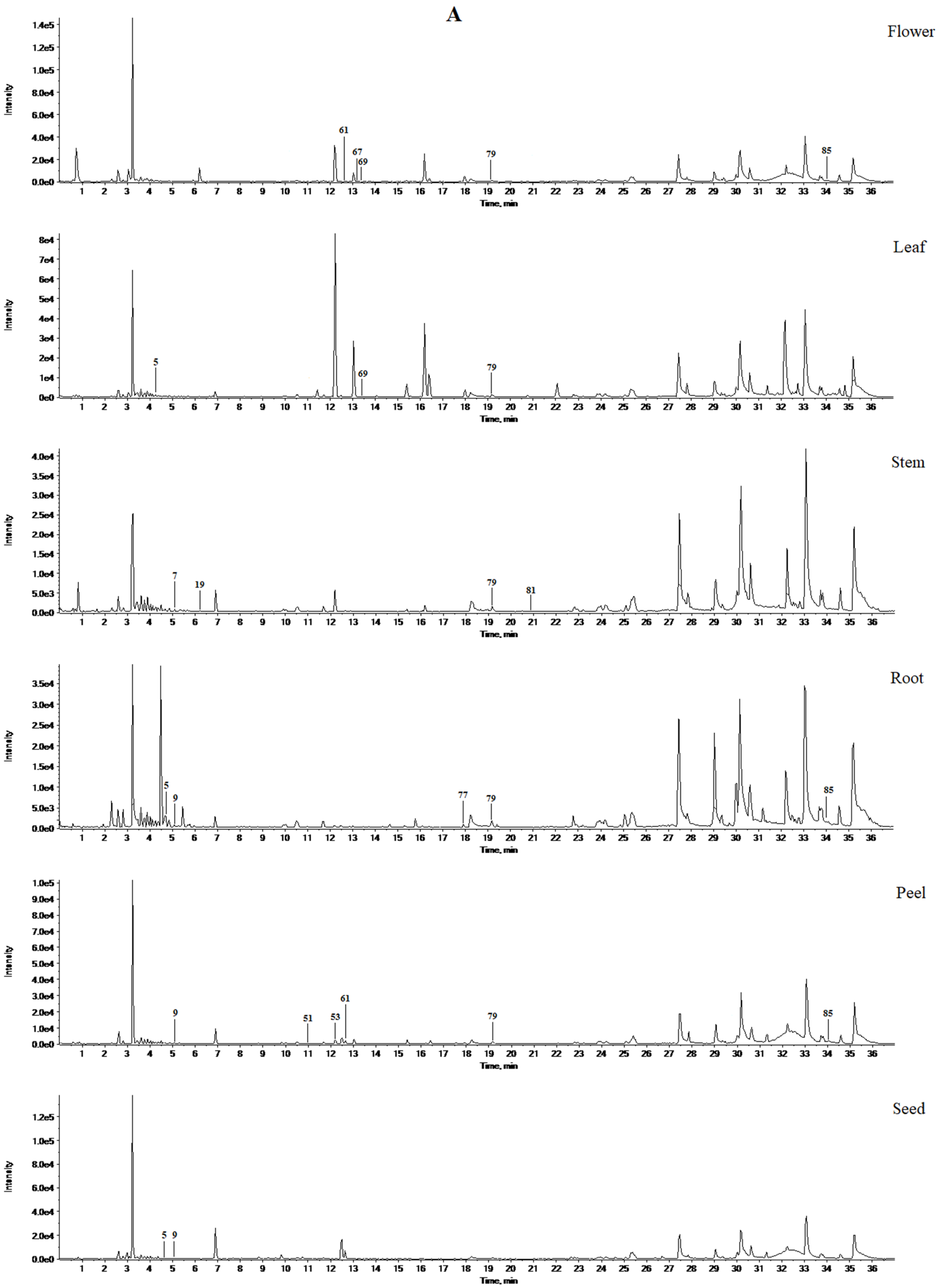
A
Flower
Leaf
Stem
Root
Peel
Seed
Intensity
Time, min
61
67
69
79
85
5
7
9
19
51
53
77
81

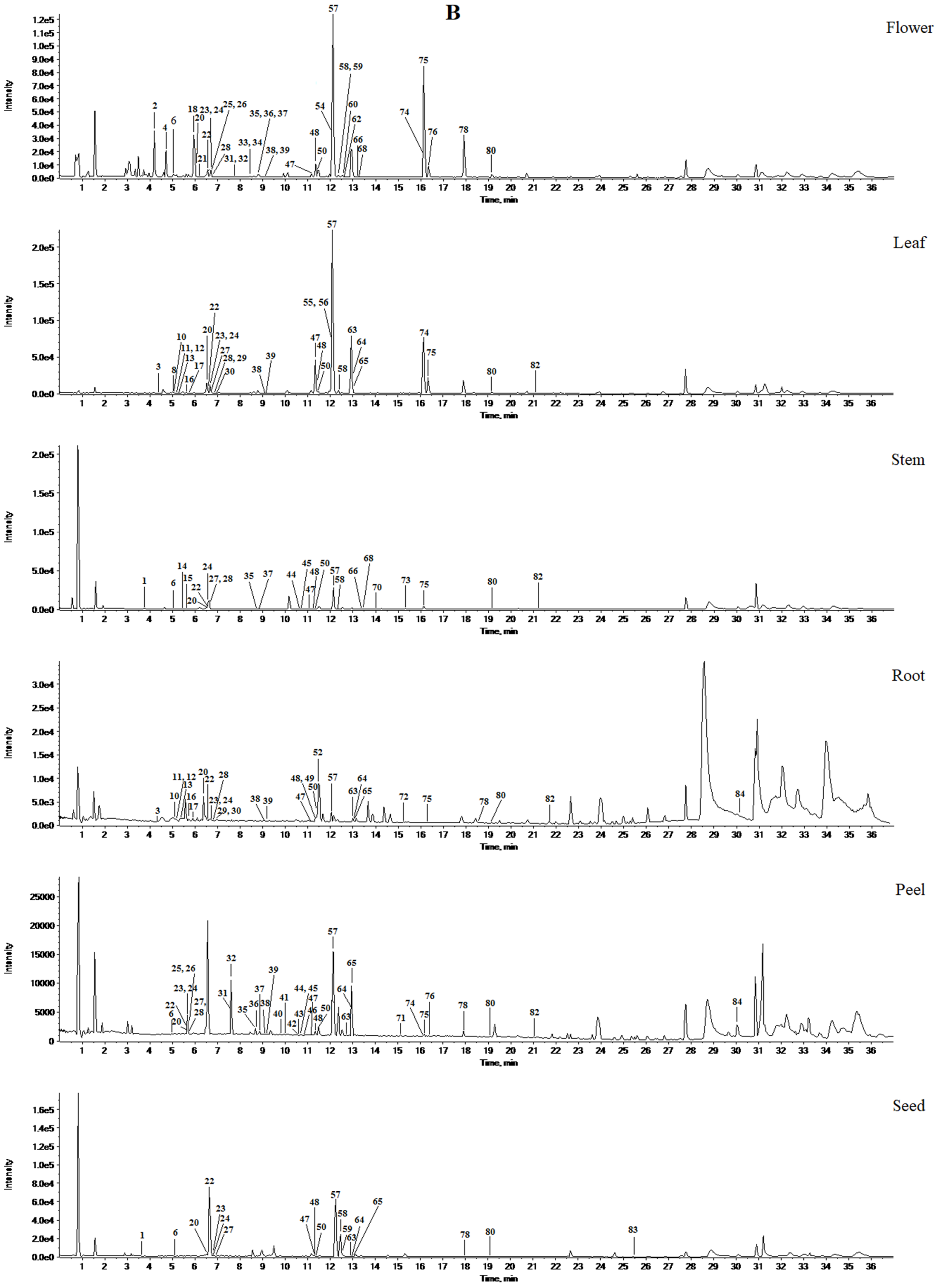

Figure S1. The BPC graphs of six parts of *Datura metel* L. (*D. metel* L.): 1A for ESC$^+$, 1B for ESC$^-$.

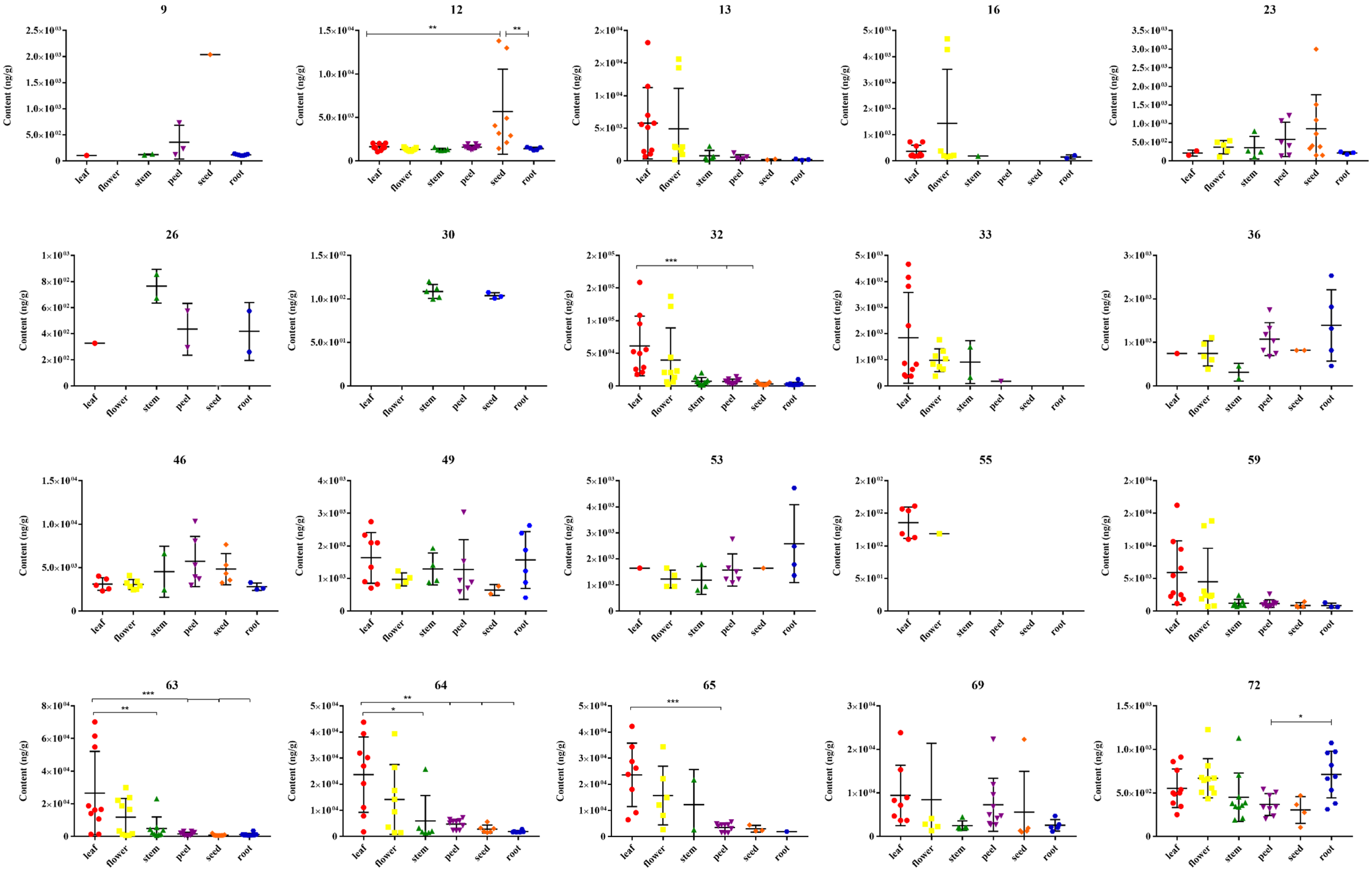
9
12
13
16
23
26
30
32
33
36
46
49
53
55
59
63
64
65
69
72
Content (ng/g)
leaf
flower
stem
peel
seed
root

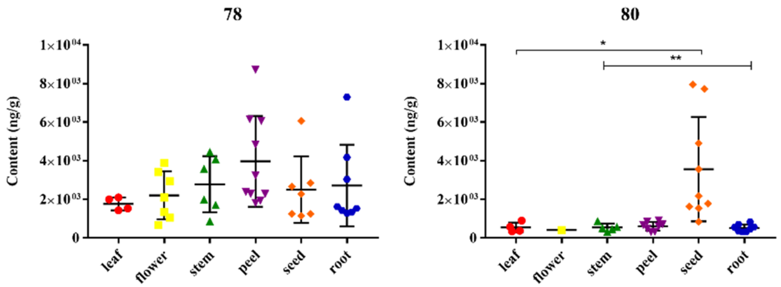

* P<0.05; ** P<0.01; *** P<0.005

Figure S2. The distribution of 22 withanolides in six parts of *D. metel* L..: Baimantuoluoside H (9), Daturafoliside K (12), Baimantuoluoside B (13), Daturafoliside B (16), Daturafoliside A (23), 5α,12α,27-trihydroxy-(20S,22R)-6α,7α-epoxy-1-oxowitha-2,24-dienolide (26), Daturafoliside O (30), Daturametelin J (32), Daturafoliside Q (33), Daturafoliside D (36), Daturafoliside S (46), Daturafoliside I (49), 7α,27-dihydroxy-(20S,22R)-1-oxowitha-2,5,24-trienolide-27-O-β-D-glucopyranosy (53), Daturataturin B (55), Daturafoliside Y (59), 7α,27-dihydroxy-(20S, 22R)-7-methoxy-1-oxowitha-3,5,24-trienolide-27-O-β-D-glucopyranosy (63). Daturametelin I (64), Daturataturin A (65), 7α,27-dihydroxy-1-oxowitha-2,5,24-trienolide (69), Daturafoliside F (72), Daturafoliside X (78), Daturametelin A (80).

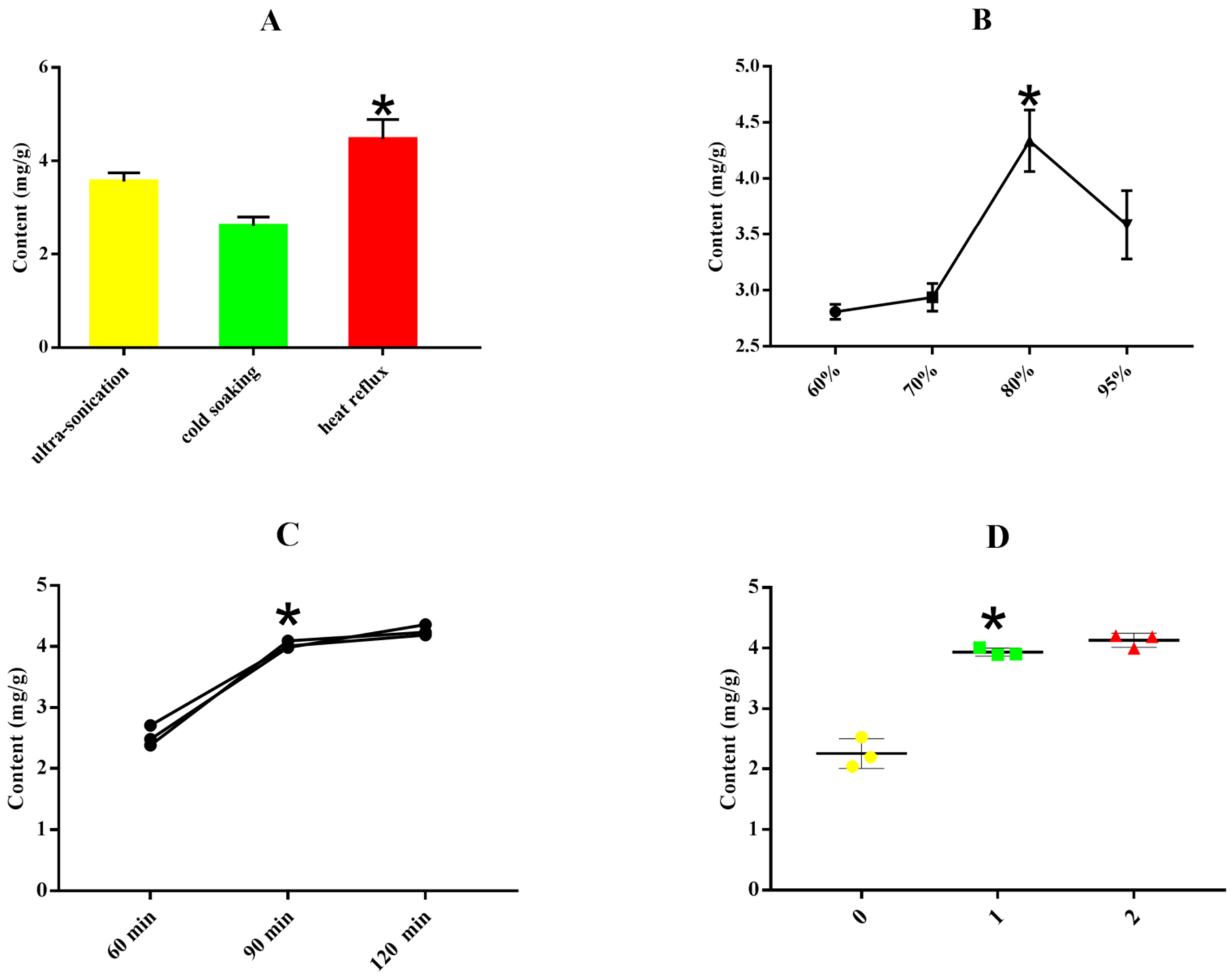

Figure S3. The total withanolides contents of different extraction conditions: extraction methods (A), extraction solvents (B), extraction times (C) and extraction repeats (D).

A (extraction solvent: 80% ethanol; extraction times: 90 min; extraction repeats: 1)

B (extraction method: heat reflux; extraction times: 90 min; extraction repeats: 1)

C (extraction method: heat reflux; extraction solvent: 80% ethanol; extraction repeats: 1)

D (extraction method: heat reflux; extraction solvent: 80% ethanol; extraction times: 90 min)

** The best choice of each extraction condition.*

9

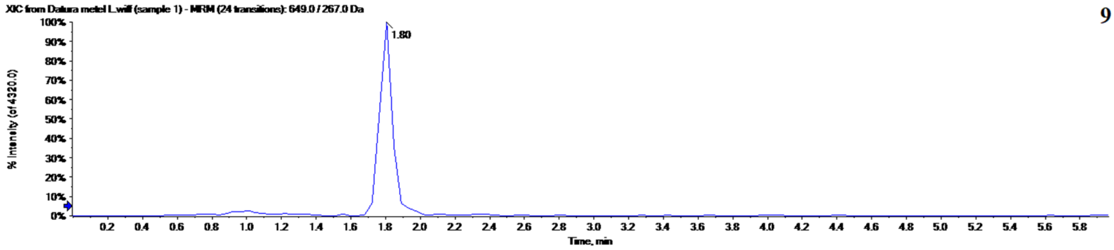

12

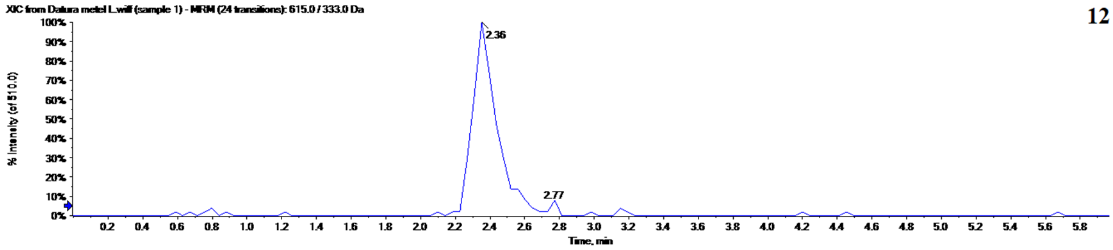

13

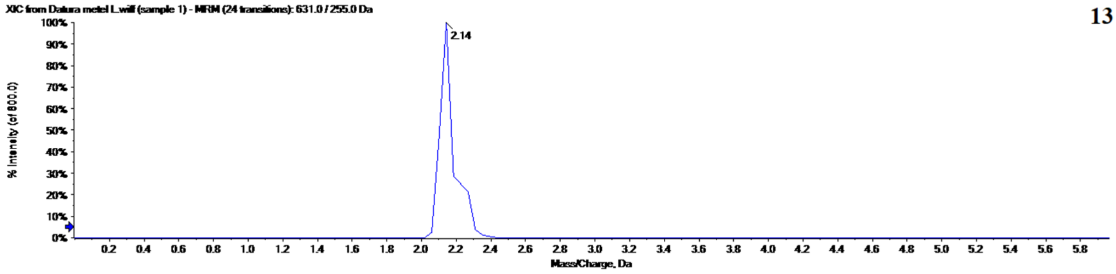

16

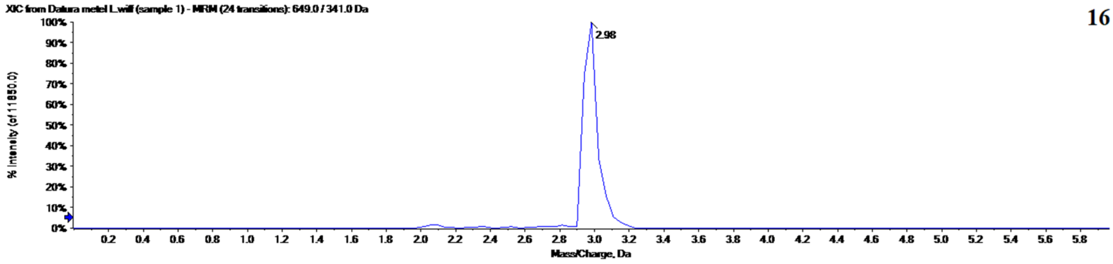

23

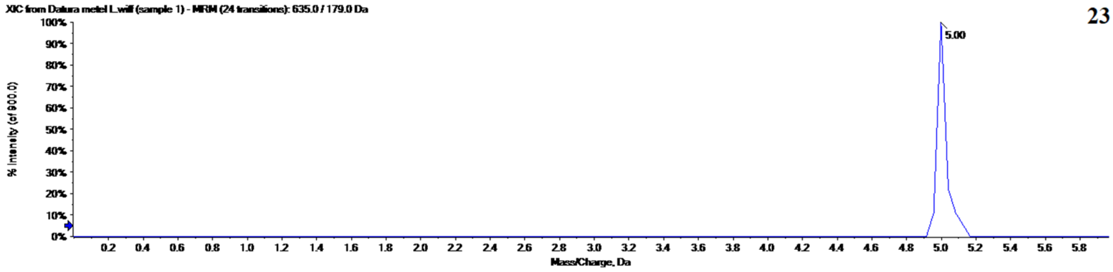

26

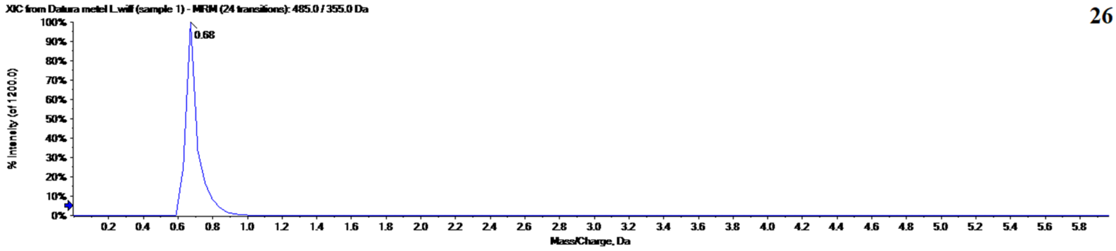

30

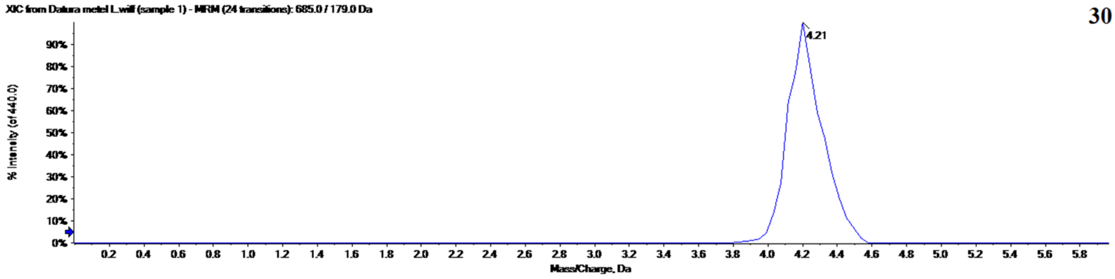

32

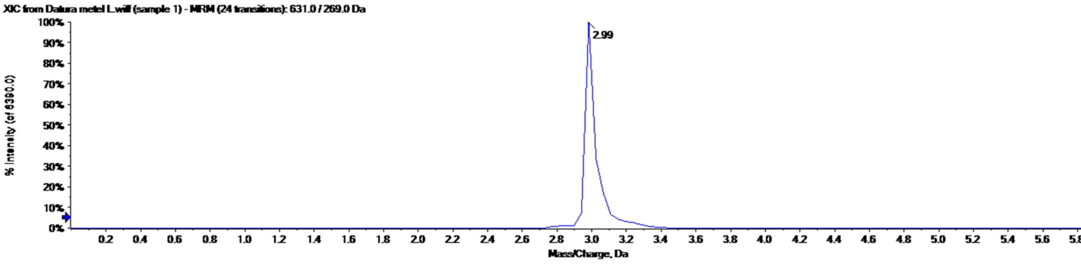

33

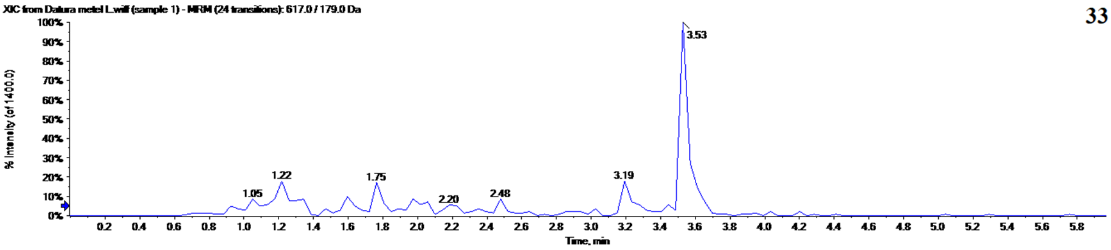

36

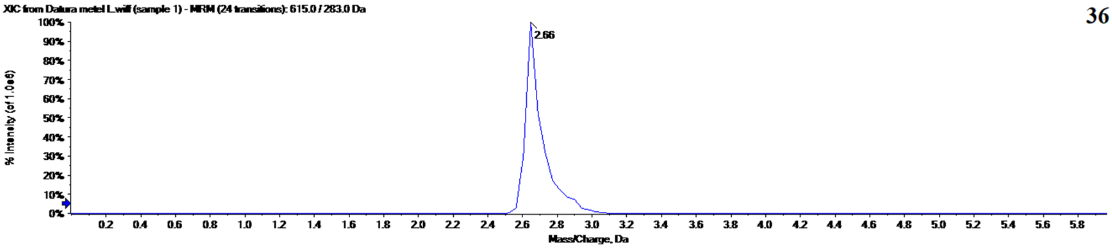

46

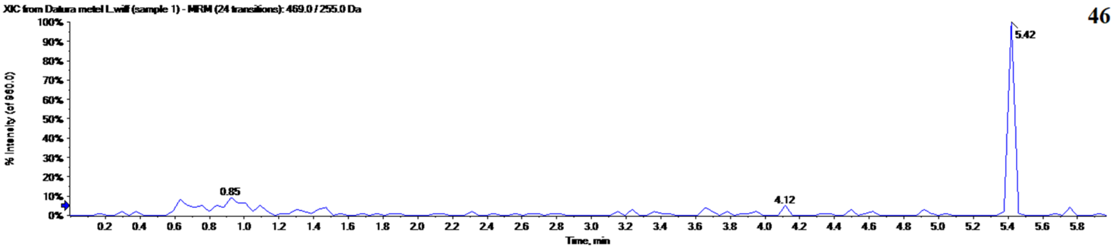

49

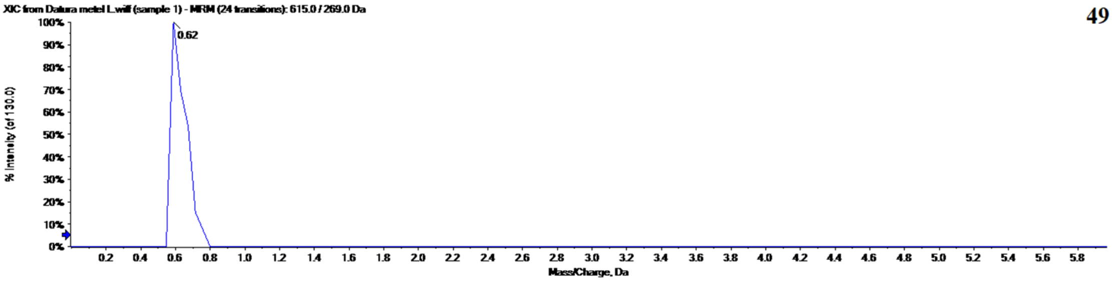

53

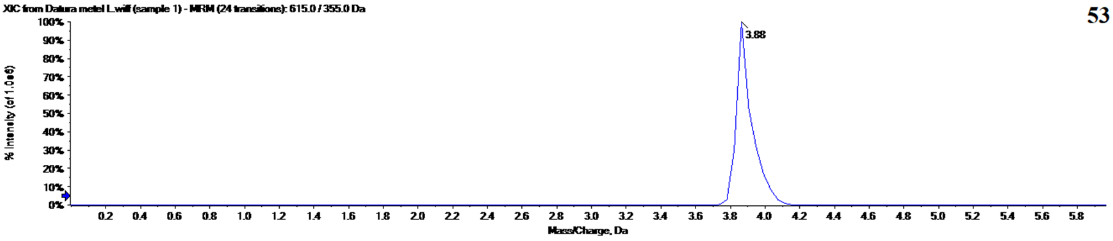

55

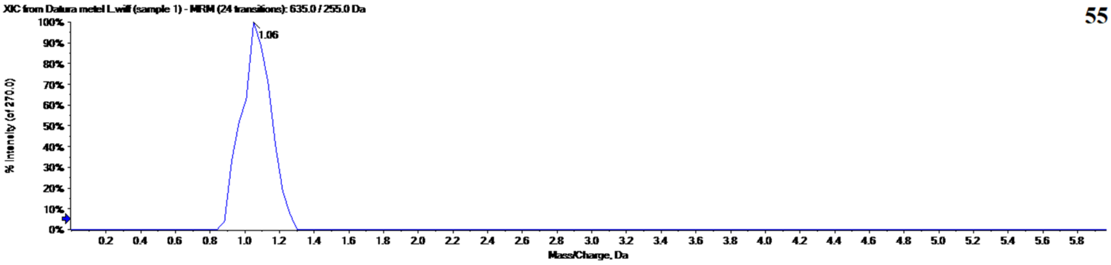

59

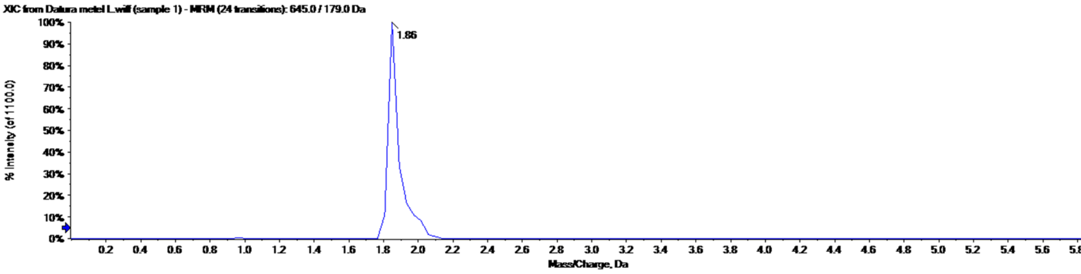

63

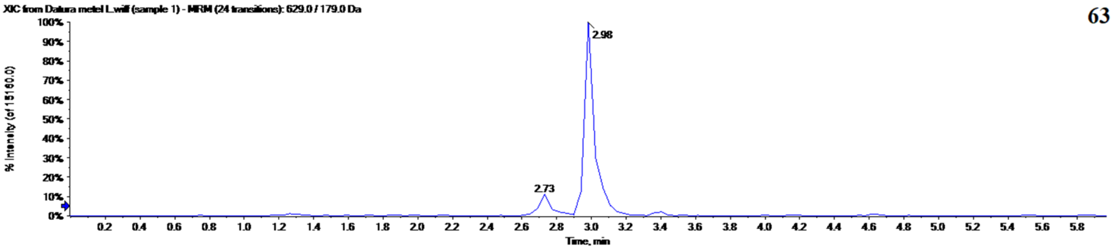

64

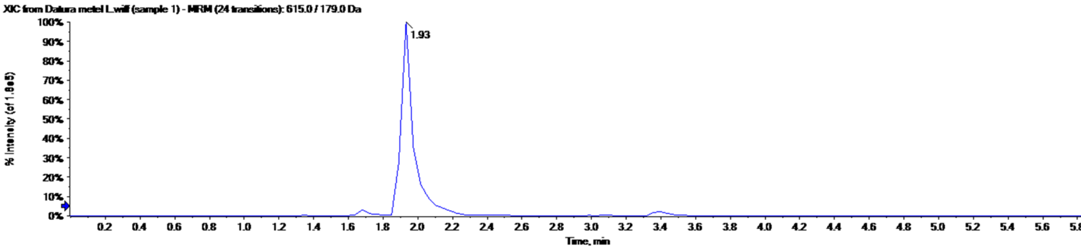

65

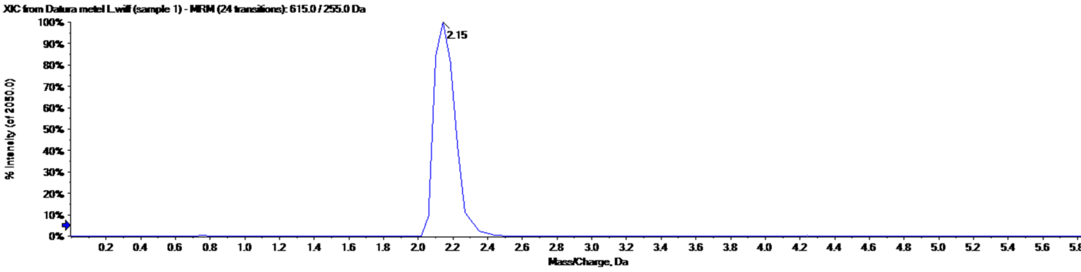

69

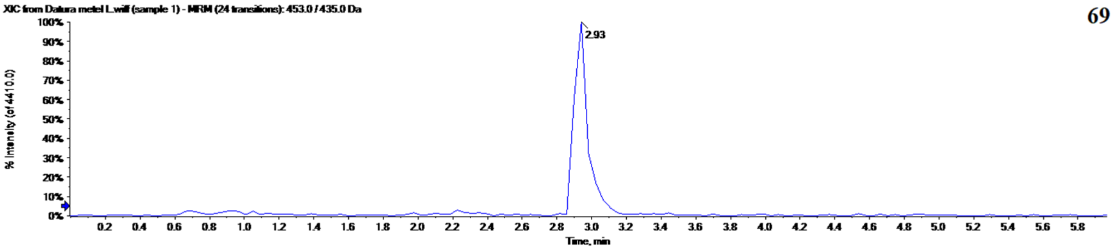

72

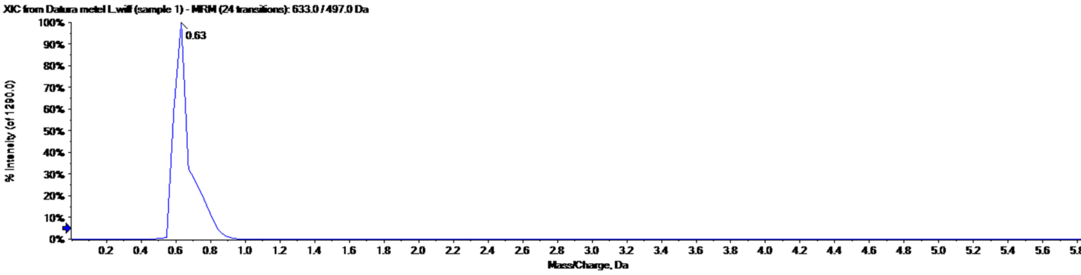

78

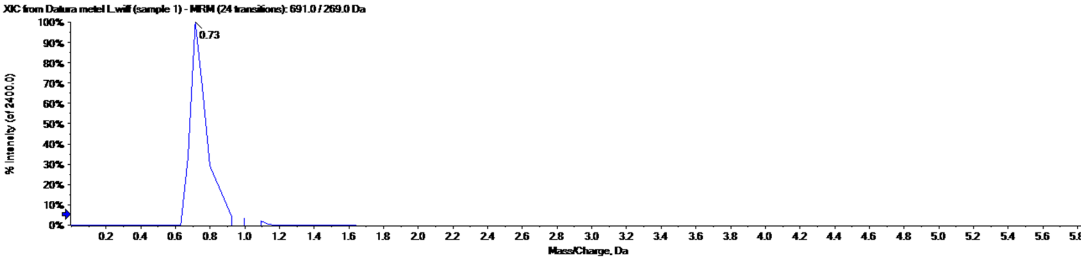

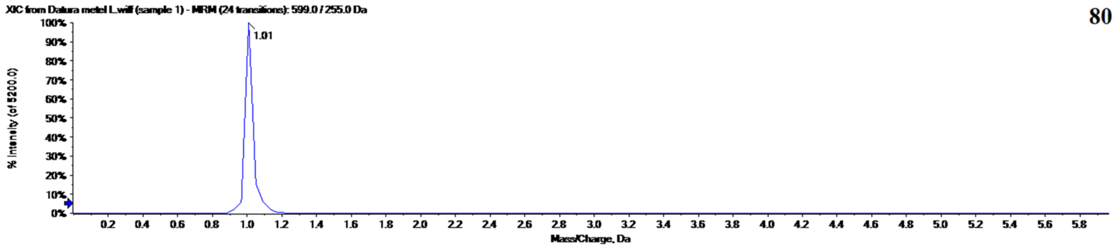

Figure S4. The MRM chromatograms of 22 bioactive withanolides: Baimantuoluoside H (9), Daturafoliside K (12), Baimantuoluoside B (13), Daturafoliside B (16), Daturafoliside A (23), 5α,12α,27-trihydroxy-(20S,22R)-6α,7α-epoxy-1-oxowitha-2,24-dienolide (26), Daturafoliside O (30), Daturametelin J (32), Daturafoliside Q (33), Daturafoliside D (36), Daturafoliside S (46), Daturafoliside I (49), 7α,27-dihydroxy-(20S,22R)-1-oxowitha-2,5,24-trienolide-27-O-β-D-glucopyranosy (53), Daturataturin B (55), Daturafoliside Y (59), 7α,27-dihydroxy-(20S,22R)-7-methoxy-1-oxowitha-3,5,24-trienolide-27-O-β-D-glucopyranosy (63). Daturametelin I (64), Daturataturin A (65), 7α,27-dihydroxy-1-oxowitha-2,5,24-trienolide (69), Daturafoliside F (72), Daturafoliside X (78), Daturametelin A (80).

Table S1. The contents of 22 bioactive withanolides in *Datura metel* L. (*D. metel* L.).

Source (ng/g)	S1-flower	S1-seed	S1-root	S1-peel	S1-leaf	S1-stem	S2-flower	S2-seed	S2-root	S2-peel	S2-leaf	S2-stem
9	-	-	140.233	728.016	-	-	-	-	112.71	-	-	129.405
12	1519.245	4045.935	-	1953.281	1674.26	1178.221	1100.713	-	-	1448.115	1038.705	-
13	192.896	-	107.993	-	1056.007	-	-	-	-	-	18140.379	419.315
16	-	-	-	-	258.339	-	-	-	-	-	727.876	-
23	266.17	2999.298	-	495.831	-	266.17	105.385	381.005	-	-	-	-
26	-	-	259.601	292.719	-	673.697	-	-	-	-	-	-
30	-	102.993	-	-	-	100.043	-	-	-	-	-	-
32	6221.529	1788.718	2008.761	2731.818	28416.033	2165.935	2999.037	1809.675	1411.461	4999.331	158700.01	5236.296
33	-	-	-	-	424.152	-	641.815	-	-	-	831.781	-
36	-	-	-	1030.941	-	458.022	386.382	-	2535.004	-	-	171.492
46	3419.541	7673.484	-	8072.468	2322.89	-	2821.387	-	3286.682	-	-	2455.854
49	-	-	1869.866	1520.236	1345.317	-	1019.866	-	879.047	--	2103.087	-
53	-	-	-	1222.518	-	942.418	942.251	-	4724.88	-	-	-
55	-	-	-	-	110.448	-	-	-	-	-	-	-
59	694.48	-	-	-	2784.945	-	-	-	-	771.813	16235.135	683.425
63	755.043	629.758	672.116	642.11	16226.221	1390.303	570.644	581.23	575.937	3105.584	1292.806	-
64	1439.835	-	-	2248.415	43774.835	-	-	-	-	5964.329	-	-
65	-	-	-	1414.541	42201.411	-	-	-	-	4492.691	-	-
69	-	-	-	4174.614	8949.725	-	-	1268.133	-	4710.351	-	-
72	555.077	104.177	-	338.208	250.183	338.205	660.26	-	312.451	202.287	499.242	344.644
78	3889.306	2660.461	7292.813	8710.686	-	-	2943.938	2849.584	-	1933.095	-	4078.485
80	-	7943.392	459.588	704.705	369.712	-	410.554	1538.07	573.981	-	337.018	868.097

Source (ng/g)	S3-flower	S3-seed	S3-root	S3-peel	S3-leaf	S3-stem	S4-flower	S4-seed	S4-root	S4-peel	S4-leaf	S4-stem
9	-	-	100.014	-	-	-	-	-	123.991	-	-	-
12	1348.733	3162.39	1426.235	1503.752	1209.215	1317.741	1599.148	1427.462	1565.736	1427.462	1534.735	-
13	2136.041	-	-	-	5126.193	145.727	2187.968	273.962	-	397.706	6980.069	-
16	185.903	-	97.343	-	203.388	-	199.522	-	-	-	224.507	-
23	-	1093.002	-	-	-	-	541.762	403.95	-	403.95	-	-
26	-	-	-	-	-	-	-	-	-	-	-	-
30	-	-	-	-	-	111.981	-	-	-	-	-	108.759
32	20692.445	3591.117	3633.028	4649.437	49656.729	6933.766	22849.528	7004.449	1872.529	7004.449	52446.634	6001.164
33	1137.562	-	-	181.322	3817.961	-	1326.798	-	-	-	4662.612	-
36	-	-	458.008	-	-	-	1102.538	816.05	-	816.05	-	-
46	-	4316.917	-	-	-	-	2887.83	5280.591	-	5280.591	4017.735	-
49	1228.793	-	-	-	-	1403.631	-	-	-	937.315	2336.216	1928.087
53	-	-	1362.54	-	-	802.312	1362.707	1642.835	-	1642.835	-	-
55	-	-	-	-	156.767	-	-	-	-	-	161.298	-
59	2425.319	680.365	636.797	782.83	5437.102	921.521	3058.097	1449.177	-	1244.922	6566.228	917.821
63	23752.564	863.995	1292.351	3141.572	54831.857	4459.723	29880.518	1957.605	845.039	1957.605	70133.8	3828.531
64	14177.25	3009.985	2753.126	5775.459	26989.474	1410.943	17618.866	5601.791	1578.26	5601.791	31951.731	1718.767
65	12047.366	2133.642	1929.455	4535.193	23828.705	-	14958.713	4441.68	-	4441.68	28719.891	-
69	-	1020.988	1167.583	4957.443	7182.967	1817.139	4102.77	22306.143	2006.925	22305.614	23827.078	2064.284
72	810.568	275.946	819.164	232.999	383.307	357.539	1229.244	469.171	973.755	469.171	345.824	381.154
78	-	-	1336.868	2376.909	-	1715.113	-	6063.893	-	6063.893	2093.226	4456.654
80	-	1627.946	320.694	-	-	-	-	835.418	-	835.418	-	-

Source (ng/g)	S5-flower	S5-seed	S5-root	S5-peel	S5-leaf	S5-stem	S6-flower	S6-seed	S6-root	S6-peel	S6-leaf	S6-stem
9	-	-	114.708	-	-	116.256	-	-	103.881	-	106.203	-
12	1147.208	4898.527	1542.984	1596.74	1532.543	1565.742	1286.718	-	1271.21	1674.251	2046.291	-
13	999.397	-	-	-	5753.509	485.335	15607.535	-	-	626.839	5573.1	-
16	150.479	-	-	-	190.044	-	4675.935	-	-	-	728.372	-
23	-	335.087	-	-	-	-	-	-	174.294	1069.974	-	-
26	-	-	-	-	-	-	-	-	-	-	-	-
30	-	-	-	-	-	120.234	-	100.752	-	-	-	102.055
32	13388.383	2648.01	2218.361	4607.617	55556.692	13535.986	122011.31	-	2124.024	7372.417	108386.1	5917.592
33	1032.047	-	-	-	4160.419	333.712	729.722	-	-	-	865.403	-
36	959.372	-	-	-	-	-	601.245	-	816.121	1317.442	744.496	-
46	-	-	-	3685.43	-	-	4084.23	-	2489.03	2954.285	2555.525	-
49	879.047	-	-	879.059	899.392	879.036	762.442	-	1228.793	704.151	820.745	-
53	-	-	-	-	-	-	-	-	1782.857	1082.44	-	-
55	-	-	-	-	154.141	-	-	-	-	-	-	-
59	1855.351	-	-	619.622	1180.991	1466.365	13105.814	-	-	951.125	10697.108	893.281
63	16498.582	625.345	723.28	1462.629	61518.362	5534.465	1632.907	-	1142.377	642.547	1349.449	1177.77
64	9477.891	1529.585	1879.317	2357.114	30192.66	3214.16	1441.397	-	-	5140.028	1750.679	1467.375
65	8074.247	-	-	1528.628	26176.599	2609.992	-	-	-	3921.132	-	-
69	2272.309	1314.76	4714.897	2991.542	8287.323	1979.012	-	-	-	6809.738	-	-
72	671.022	-	960.879	542.178	537.9	1130.491	645.238	365.944	688.193	-	486.364	454.16
78	-	1242.457	4172.858	2282.329	-	3583.455	1336.962	1147.858	3038.593	2282.292	1998.758	1998.646
80	-	3564.36	-	598.637	-	-	-	-	557.623	663.857	-	508.61

Source (ng/g)	S7-flower	S7-seed	S7-root	S7-peel	S7-leaf	S7-stem	S8-flower	S8-seed	S8-root	S8-peel	S8-leaf	S8-stem
9	-	-	-	-	-	-	-	-	-	121.671	-	-
12	1178.224	2898.881	-	1255.72	2015.259	-	-	2108.3	-	1317.704	-	1255.72
13	-	-	291.966	1211.738	11437.53	664.596	1645.527	-	-	361.479	1645.527	-
16	-	-	206.226	-	566.7	-	210.884	-	-	-	210.884	-
23	-	725.538	220.23	1207.768	266.156	243.202	426.936	1506.411	243.198	151.33	151.34	-
26	-	-	-	-	-	-	-	-	-	574.315	325.856	855.842
30	-	107.572	-	-	-	-	-	-	-	-	-	-
32	6441.479	3067.139	10234.668	14010.81	95076.878	8563.22	20986.404	2103.081	1898.726	10707.413	20986.404	1720.579
33	-	-	-	-	2299.821	-	372.237	-	-	-	372.237	-
36	672.842	-	-	1174.234	-	-	-	815.965	-	744.439	-	-
46	2489.03	3253.455	-	10331.751	3685.351	-	-	3552.532	2621.98	-	-	-
49	-	-	-	3030.874	2744.23	-	-	-	412.72	-	-	937.35
53	942.362	-	-	1502.618	-	-	1642.918	-	-	1222.518	1642.918	1782.968
55	-	-	-	-	113.078	-	118.969	-	-	-	118.969	-
59	800.011	606.118	1290.885	2615.6	9487.642	1174.272	2251.738	-	-	1285.237	2251.738	-
63	3381.551	608.58	1151.164	680.052	16575.249	2319.307	18754.14	587.406	684.452	1592.788	18754.14	1456.888
64	3591.639	1903.212	1638.336	6556.343	20285.325	2019.98	39348.784	1765.724	1752.606	7221.136	39348.784	-
65	2668.56	-	-	4868.095	18063.391	-	34354.422	-	-	5651.398	34354.422	-
69	1365.405	-	-	9565.606	4681.011	-	2840.294	-	2136.181	11924.984	3699.038	2034.997
72	677.476	-	533.585	316.745	859.955	705.354	499.242	-	379.339	507.84	499.242	211.538
78	3416.575	-	1431.373	3227.641	1431.467	864.269	675.146	2282.329	1620.664	4834.824	-	-
80	-	4904.198	353.376	320.684	-	573.981	-	7722.949	704.71	904.868	892.609	312.513

Source (ng/g)	S9-flower	S9-seed	S9-root	S9-peel	S9-leaf	S9-stem	S10-flower	S10-seed	S10-root	S10-peel	S10-leaf	S10-stem
9	-	2035.829	-	231.495	-	-	-	-	129.403	-	-	-
12	1426.238	13812.043	1209.229	1953.275	1999.772	-	1286.715	12990.344	-	1550.243	1534.753	1224.706
13	2041.706	-	211.738	-	1438.114	-	14280.664	155.169	-	174.033	673.97	2230.385
16	376.43	-	-	-	176.365	-	4269.682	-	-	-	-	186.179
23	495.84	151.344	-	128.367	-	105.408	-	151.34	-	-	-	794.414
26	-	-	574.315	-	-	-	-	-	-	-	-	-
30	-	-	-	-	-	-	-	-	-	-	-	-
32	43673.61	2176.411	3161.401	3313.494	25557.108	1987.797	137241.45	5791.609	2540.691	9726.581	17790.279	20329.467
33	1770.652	-	-	-	635.164	-	840.286	-	-	-	376.413	1490.918
36	-	-	1317.499	1747.209	-	-	-	-	1818.764	672.842	-	-
46	3220.24	-	-	3910.061	2954.312	6609.889	2455.854	-	-	-	-	-
49	-	762.512	2627.694	-	2101.059	-	-	529.336	2394.519	587.627	704.163	-
53	-	-	2483.524	2763.624	-	-	-	-	-	-	-	-
55	-	-	-	-	-	-	-	-	-	-	-	-
59	2415.483	-	638.63	904.333	2487.204	-	13852.235	792.645	-	1185.325	1822.242	2402.045
63	22152.004	585.642	3483.914	581.231	14120.497	657.107	1213.373	1060.914	605.934	2426.081	10720.882	23077.262
64	26431.402	-	1689.146	2366.068	11102.129	-	-	3249.612	-	4154.917	7856.255	25779.624
65	22184.353	-	-	1446.022	9138.604	-	-	2370.034	-	3009.424	6423.691	21739.793
69	31591.404	-	2739.163	2698.351	15332.153	-	-	1920.332	-	2704.377	3691.161	4476.422
72	434.828	-	1074.663	-	761.196	190.07	505.682	-	664.579	333.922	911.485	389.021
78	2093.282	1242.476	1526.009	6158.192	1525.934	-	1053.316	-	1289.662	1809.711	-	-
80	-	2167.188	827.247	304.355	-	-	-	1775.019	316.601	467.757	573.968	459.571

- Not detected

Table S2. The total withanolides contents of different extraction conditions for *D. metel* L.

Extraction methods (mg/g)	Content (mg/g)			$\bar{x}$±*s* (mg/g)
	1	2	3	
Ultra-sonication	3.584	3.369	3.738	3.56±0.19
Cold soaking	2.487	2.498	2.833	2.61±0.2
Heat reflux	4.385	4.923	4.099	4.47±0.42
Extraction solvents (mg/g)	Content (mg/g)			$\bar{x}$±*s* (mg/g)
	1	2	3	
60% Ethanol	2.840	2.856	2.733	2.81±0.07
70% Ethanol	3.034	2.983	2.799	2.94±0.12
80% Ethanol	4.038	4.398	4.576	4.34±0.27
95% Ethanol	3.899	3.287	3.576	3.59±0.31
Extraction times (mg/g)	Content (mg/g)			$\bar{x}$±*s* (mg/g)
	1	2	3	
60 min	2.385	2.486	2.711	2.53±0.17
90 min	4.092	3.985	4.010	4.03±0.06
120 min	4.234	4.361	4.188	4.26±0.09
Extraction repeats (mg/g)	Content (mg/g)			$\bar{x}$±*s* (mg/g)
	1	2	3	
0	2.041	2.532	2.198	2.26±0.25
1	3.892	3.901	4.011	3.93±0.07
2	4.208	4.189	3.998	4.13±0.12

Table S3. Information of *D. metel* L. materials

Producing areas No.	Locality	Coordinate	Positions (Harvest time)
S1	Fuyang, Anhui	115°42′55″N 32°56′54″E	Flower, Root, Leaf and Stem (2019, 07); Peel and Seed (2019, 08)
S2	Haikou, Hainan	109°56′53″N 19°55′28″E	Flower, Root, Leaf and Stem (2019, 06); Peel and Seed (2019, 07)
S3	Xingtai, Hebei	114°23′18″N 37°12′40″E	Flower, Root, Leaf and Stem (2019, 07); Peel and Seed (2019, 08)
S4	Dujiangyan, Sichuan	103°39′12″N 31°27′94″E	Flower, Root, Leaf and Stem (2019, 06); Peel and Seed (2019, 07)
S5	Harbin, Heilongjiang	126°39′18″N 45°44′18″E	Flower, Root, Leaf and Stem (2019, 08); Peel and Seed (2019, 09)
S6	Ganzhou, Jiangxi	114°48′40″N 25°48′14″E	Flower, Root, Leaf and Stem (2019, 07); Peel and Seed (2019, 08)
S7	Zhaotong, Yunnan	103°53′23″N 27°18′30″E	Flower, Root, Leaf and Stem (2019, 06); Peel and Seed (2019, 07)
S8	Baotou, Neimenggu	109°41′10″N 40°43′28″E	Flower, Root, Leaf and Stem (2019, 07); Peel and Seed (2019, 08)
S9	Baoji, Shanxi	107°11′36″N 34°17′34″E	Flower, Root, Leaf and Stem (2019, 07); Peel and Seed (2019, 08)
S10	Jinhua, Zhejiang	119°31′58″N 29°23′17″E	Flower, Root, Leaf and Stem (2019, 07); Peel and Seed (2019, 08)

Table S4. UPLC-Q-TRAP-MS/MS detection parameters for 22 bioactive withanolides in *D. metel* L.

Peak No.	Compound	Precursor ion / Product ion	CE	DP	t_R (min)
9	Baimantuoluoside H	649/267	-20	-70	1.80
12	Daturafoliside K	615/333	-21	-70	2.36
13	Baimantuoluoside B	631/255	-10	-54	2.14
16	Daturafoliside B	649/341	-16	-88	2.98
23	Daturafoliside A	635/179	-13	-49	5.00
26	5α,12α,27-trihydroxy-(20S,22R)-6α,7α-epoxy-1- oxowitha-2,24-dienolide	485/355	-39	-158	0.68
30	Daturafoliside O	685/179	-16	-63	4.21
32	Daturametelin J	631/269	-16	-110	2.99
33	Daturafoliside Q	617/179	-14	-90	3.53
36	Daturafoliside D	615/283	-15	-79	2.66
46	Daturafoliside S	469/255	-19	-49	5.42
49	Daturafoliside I	615/269	-22	-75	0.62
53	7α,27-dihydroxy-(20S,22R)-1-oxowitha-2,5,24-trienolide-27-O-β-D-glucopyranosy	615/355	-15	-79	3.88
55	Daturataturin B	635/255	-12	-121	1.06
59	Daturafoliside Y	649/179	-14	-104	1.86
63	7α,27-dihydroxy-(20S,22R)-7-methoxy-1-oxowitha-3,5,24-trienolide-27-O-β-D-glucopyranosy	629/179	-16	-80	2.98
64	Daturametelin I	615/179	-19	-95	1.93
65	Daturataturin A	615/255	-12	-112	2.15
69	7α,27-dihydroxy-1-oxowitha-2,5,24-trienolide	453/435	-12	-69	2.93
72	Daturafoliside F	633/497	-15	-66	0.63
78	Daturafoliside X	691/269	-17	-67	0.73
80	Daturametelin A	599/255	-18	-70	1.01
